# Beneficial Impact of Interspecies Chimeric Renal Organoids Against a Xenogeneic Immune Response

**DOI:** 10.3389/fimmu.2022.848433

**Published:** 2022-02-15

**Authors:** Yatsumu Saito, Naoto Matsumoto, Shuichiro Yamanaka, Takashi Yokoo, Eiji Kobayashi

**Affiliations:** ^1^ Division of Nephrology and Hypertension, Department of Internal Medicine, The Jikei University School of Medicine, Tokyo, Japan; ^2^ Department of Kidney Regenerative Medicine, The Jikei University School of Medicine, Tokyo, Japan

**Keywords:** organoid, chimera, regeneration, xenotransplantation, progenitors

## Abstract

**Background:**

Animal fetal kidneys have the potential to be used as scaffolds for organ regeneration. We generated interspecies chimeric renal organoids by adding heterologous rat renal progenitor cells to single cells from mouse fetal kidneys and applying the renal development mechanism of mouse fetuses to rat renal progenitor cells to examine whether rat renal progenitor cells can differentiate into renal tissues of the three progenitor cell lineages of kidneys between different species. Furthermore, we investigated whether chimeric renal organoids with an increased proportion of recipient cells reduce xenogeneic rejection.

**Methods:**

C57BL/6JJmsSlc mice (B6 mice) and Sprague-Dawley-Tg (CAG-EGFP) rat (GFP rats) fetuses were used as donors, and mature male NOD/Shi-scid, IL-2RγKO Jic mice (NOG mice) and Sprague-Dawley rats (SD rats) were used as recipients. First, fetal kidneys were removed from E13.5 B6 mice or E15.5 GFP rats and enzymatically dissociated into single cells. These cells were then mixed in equal proportions to produce chimeric renal organoids *in vitro*. The chimeric organoids were transplanted under the renal capsule of NOG mice, and maturation of the renal tissues in the organoids was observed histologically. Furthermore, chimeric organoids were prepared by changing the ratio of cells derived from mouse and rat fetal kidneys and transplanted under the renal capsule of SD rats subjected to mild immunosuppression to pathologically analyze the strength of the xenogeneic immune response.

**Results:**

The cap mesenchyme was reconstructed *in vitro*, and nephron progenitor cells and ureteric buds were mosaically comprised GFP-negative mouse and GFP-positive rat cells. In the *in vivo* environment of immunodeficient mice, chimeric renal organoids mosaically differentiated and matured into renal tissues of three lineages. Chimeric renal organoids with high rates of recipient rat cells showed milder rejection than complete xenograft organoids. The vessels of recipient rats entered from the periphery of the transplanted chimeric renal organoids, which might reduce their immunogenicity.

**Conclusion:**

Interspecies chimeric renal organoids may differentiate into mature renal tissues of each renal progenitor cell lineage. Furthermore, they may reduce transplant rejection compared with xenograft organoids.

## Introduction

In recent years, significant advancements have been made in human stem cell research and the generation of miniature organs such as brains, eye cups, and kidneys—known as “organoids,”—from human embryonic stem cells and induced pluripotent stem cells (iPSCs) *in vitro* has garnered much scientific attention (1). However, not all complex developmental mechanisms can be faithfully reproduced *in vitro*, and there remain some challenges that need to be overcome before the cells can mature into a tissue population known as an “organ.” We aimed to generate a mature human kidney by injecting human renal progenitor cells into the nephrogenic zone (NZ) of a heterologous animal fetal kidney using the nephrogenic mechanism of the animal fetus (2–4). When animal fetal kidneys are dissociated into single cells and then reaggregated *in vitro* in a mouse model, renal organoids containing nephrons and collecting ducts are formed (5); this indicates that the cells comprising animal fetal kidneys already have the ability to form mature renal tissues. Furthermore, chimeric aggregates can be formed between different species by mixing chondrocytes from chicken and mouse fetal organs to generate organoids by reaggregating fetal-derived cells *in vitro* (6). Using this interspecies chimeric organoid phenomenon, we believe that it will soon be possible to generate kidneys with mature human renal tissue by aggregating organoids produced from fetal kidneys of different species such as pigs with renal progenitor cells derived from human iPSCs and applying the renal developmental signaling pathway of animal fetuses to human renal progenitor cells.

The kidney has three major progenitor cell types: nephron progenitor cells (NPCs), stromal progenitor cells (SPCs), and ureteric buds (UBs). In the NZ under the embryonic renal capsule, these three progenitor cells interact with one another to spontaneously differentiate into tissues of their own lineage (7). However, it is unclear whether the three progenitor cells of different species can actually form a developmental environment (niche) in the interspecies chimeric renal organoid and whether the three lineages—nephron, stroma, and collecting ducts—can differentiate and mature simultaneously. We previously reported the generation of chimeric kidneys with nephrons comprising 100% rat cells by transplanting heterologous rat kidney progenitor cells into the NZ of genetically engineered mice, in which six2-positive NPCs can be specifically removed by drugs, and replacing the mouse NPCs with rat NPCs using the mouse hind kidney as a scaffold (8). Furthermore, we reported that exogenous SPCs can differentiate into multiple types of renal stromal cells in the same species by transplanting mouse SPCs into the NZs of other mouse fetal kidneys (9). Therefore, in the future, it may be possible to generate kidneys with a higher chimerism rate that include the renal stroma in addition to the nephron. The immunogenicity of interspecies chimeric renal organoids with a high chimerism rate of human cells based on heterologous animal fetal kidneys compared with normal xenografts is of great importance for the clinical application of chimeric kidneys.

In this study, we first examined whether a heterologous chimeric nephrogenic niche can be generated *via* the *in vitro* association of single cells from mouse fetal kidneys and renal progenitor cells from rat fetal kidneys. Then, in immunocompromised mice, we assessed whether the heterologous nephrogenesis mechanism generates mature renal tissues of the abovementioned three lineages. Furthermore, we generated chimeric renal organoids with an increased proportion of recipient rat cells and investigated whether transplantation of the chimeric organoid into adult rats reduces xenogeneic immune rejection.

## Materials and Methods

### Research Animals

The Institutional Animal Care and Use Committee and the Safety Committee for Genetic Recombination Experiments of Jikei University School of Medicine approved the protocols for animal experiments (permission numbers 2021-017, 2019-013, R1-68). The experiments were conducted in accordance with the Guidelines for the Appropriate Conduct of Animal Experiments (2006) of the Science Council of Japan. Every effort was made to minimize animal suffering. Pregnant female C57BL/6JJmsSlc mice (B6 mice), pregnant female Sprague-Dawley-Tg (CAG-EGFP) rats (GFP rats), and mature male Sprague-Dawley rats (SD rats) were purchased from SLC (Shizuoka, Japan). Mature male NOD/Shi-scid, IL-2RγKO Jic mice (NOG mice) were purchased from CLEA (Tokyo, Japan).

### Single Cell Extraction From Fetal Kidneys

Single cells from mouse and rat fetal kidneys were obtained following a method (10) that was a partial modification of a method reported previously (11). In brief, pregnant E13.5 B6 mice or E15.5 GFP rats were anesthetized *via* the inhalation of isoflurane (2817774, Pfizer, New York, NY, USA). Fetuses were harvested and the maternal mouse or rat was immediately euthanized by injecting pentobarbital (120 mg/kg; Kyoritsu Pharma, Tokyo, Japan). Fetuses removed from the mother’s womb were immediately decapitated and euthanized. Fetal kidneys were harvested from the decapitated fetuses under an operating microscope and collected in 1.5-ml tubes containing α minimum essential medium (MEMα; 12561-056, Thermo Fisher Scientific, Waltham, MA, USA). The tube was then centrifuged at 700 g for 3 min, the supernatant was removed, and 1 ml of accutase (AT104, Innovative Cell Technologies, San Diego, CA, USA) was dispensed. The sample was vortexed and incubated at 37°C for 5 min (repeated twice), and further manual pipetting and incubation was performed again for 5 min. The cell suspension was then centrifuged at 300 g for 5 min to remove the subsequent supernatant of accutase. The pellet was resuspended in a volume of 1 ml of MEMα with 10% fetal bovine serum (FBS; Hyclone, Logan, UT, USA), 1% antibiotic-antimycotic (15240062, Thermo Fisher Scientific), and 10 µM Y2763 (257-00511, Wako, Osaka, Japan). In addition, the cells were passed through a 40 μm cell strainer (352340, Corning, NY, USA) to remove clumps of cells to obtain a single-cell suspension of mouse or rat cells. Cell counting of the single-cell suspensions was performed, and mouse and rat single-cell suspensions were placed on U-bottom 96-well low-cell-binding plates (174929, Thermo Fisher Scientific) in the cell ratios 6:0, 5:1, 3:3, 1:5, and 0:6 to a total of 2 × 10^5^ cells/well. Finally, the plates were centrifuged at 1,000 rpm for four min and incubated at 37°C in an incubator.

### *In Vitro* Culture of Renal Organoids

The single cells were confirmed to aggregate into spheroids by the next day (Day 1). After that, the medium containing Y27632 was removed, and the cells were cultured in medium containing MEMα, 10% FBS, and 1% antibiotic-antimycotic, with medium changes until Day 3. Spheroids were observed under an inverted microscope (Leica DMi1, Leica Microsystems, Wetzlar, Germany) or a fluorescence microscope (Olympus IX-71, Tokyo, Japan) and sampled on Day 4 or implanted under the renal capsule of NOG mice or SD rats. Specimens were whole-mount immunostained or frozen sectioned for fluorescent immunostaining or hematoxylin–eosin (HE) staining.

### Transplantation of Renal Organoids Under the Renal Capsule

Renal organoids were transplanted under the renal capsule using a previously described method (10). The following is a brief description of the procedure. First, the recipient was anesthetized with isoflurane inhalation and a midline abdominal incision was made. The intestine was moved to the left or right side to expose the kidney, and the renal capsule in the lower part of the kidney was dissected at nearly 1 mm using a microshear. Then, the tip of the outer cylinder of the 22G surflo (SR-OT2225C, Terumo Corp, Tokyo, Japan) was cut at an angle. While the cut surface of the outer cylinder of the surflo was facing the renal parenchyma, it was inserted through the incision in the renal capsule to avoid damaging the renal parenchyma, and a small amount of saline water was placed under the renal capsule to detach a part of the renal capsule. After that, spheres in a 96-well plate were inhaled using the outer cylinder of the surflo with a syringe attached. The outer cylinder of the same surflo was inserted into the incision, and the renal organoids were transplanted under the renal capsule. The abdomen was then closed with a 5-0 thread. The recipients were mature male NOG mice or SD rats. Recipient SD rats received a small dose of an immunosuppressive drug (tacrolimus 0.3 mg/kg/day, Astellas Pharma, Tokyo, Japan), which was rejected when MNs of B6 mice were xenotransplanted into SD rats (12). At 14 days after transplantation, recipient NOG mice or SD rats were euthanized, and the transplanted renal organoids were subsequently collected and observed under a fluorescent stereomicroscope (Leica M205FA, Leica Microsystems, Wetzlar).

### Whole-Mount Immunostaining, Immunostaining, and HE Staining of Frozen Sections

Renal organoid specimens for whole-mount immunostaining were fixed in 4% paraformaldehyde (163-20145, Wako) for 15 min at 4°C and then washed thrice with phosphate-buffered saline (PBS; 049-29793, Wako). Specimens were blocked for 1 hour at room temperature with 1% donkey serum (017-000-001, Jackson ImmunoResearch Laboratories, West Grove, PA, USA), 0.2% skim milk (190-12865, Wako), and 0.3% Triton X-100/PBS (25987-85, Nacalai Tesque, Kyoto, Japan) and were subsequently incubated with primary antibodies overnight at 4°C. After washing thrice with PBS, the samples were incubated with secondary antibodies conjugated with Alexa Fluor 488, 546, and 647 for 1 hour at room temperature. Specimens were mounted with SlowFadeTM Diamond Antifade Mountant with 4′,6-diamidino-2-phenylindole (DAPI; S36973, Invitrogen, Carlsbad, CA, USA). Specimens were observed under a fluorescence microscope (LSM880 confocal, Carl Zeiss). Specimens of renal organoids to be cryosectioned were fixed in 4% paraformaldehyde in PBS for 60 min and dehydrated in 20% sucrose in PBS. Specimens were embedded in OCT compound (4583, Sakura Finetek, Tokyo, Japan) and 8-μm thick frozen sections were prepared. HE staining was performed according to the standard procedures for histological analysis. Antigen activation for immunofluorescence staining was performed in a warm bath at 70°C for 20 min. After blocking for 1 hour at room temperature, the sections were washed thrice with PBS and then incubated with primary antibodies overnight at 4°C. The sections were then washed thrice with PBS and incubated with secondary antibodies conjugated with Alexa Fluor 488, 546, and 647 for 1 hour at room temperature. Afterward, the sections were then washed thrice with PBS and mounted using SlowFadeTM Diamond Antifade Mountant with DAPI and observed under an all-in-one fluorescence microscope (BZ-X800, Keyence, Osaka, Japan) or a fluorescence microscope (LSM880 confocal, Carl Zeiss). The primary antibodies used were as follows: chicken anti-GFP (ab13970, Abcam, Cambridge, MA, USA), rabbit anti-Six2 (11562-1-AP, ProteinTech, Rosemont, IL, USA), guinea pig anti-nephrin (GP-N2, Progen, Heidelberg, Germany), goat anti-megalin (sc-16478, Santa Cruz Biotechnology, Santa Cruz, CA, USA), mouse anti-E-cadherin (Ecad) (610181, BD, San Jose, CA, USA), rat anti-cytokeratin 8 (TROMA-I-C, DSHB, Iowa City, IA, USA), rabbit anti-platelet-derived growth factor receptor b (PDGFRb) (ab32570, Abcam), mouse anti-α SMA (A2547, Sigma-Aldrich, St. Louis, MO, USA), rabbit anti-CD31 (ab28364, Abcam), rabbit anti-NKCC2 (SPC-401D, StressMarq Bioscience), mouse anti-aquaporin 2 (sc-515770, Santa Cruz Biotechnology), rabbit anti- V-ATPase B1/2 (sc-55544, Santa Cruz Biotechnology), goat anti-GATA3 (AF2605, R&D Systems), and goat anti-renin (AF4277, R&D Systems).

### Measurement of the Chimeric Rate of the Constituent Cells of the Cap Mesenchyme

Immunostained sections of frozen organoids were photographed with a fluorescence microscope. The total number overall and the number of GFP-positive cells of Six2-positive NPCs and GATA3-positive UBs in each cap mesenchyme were counted under the same magnification, and the percentage of GFP-positive NPCs and GFP-positive UBs (%) was calculated by dividing the number of GFP-positive cells by the total number of cells. Two randomly selected sections from the renal organoid specimen were photographed at the same magnification, and a total of six images of each were made for analysis. This work was performed by two researchers.

### Measurement of the Chimeric Rate of Each of the Three Renal Lineages in Chimeric Renal Organoids

Immunostained sections of frozen organoids were photographed with a fluorescence microscope. The total number overall and the number of GFP-positive cells of GATA3-positive cells as collecting ducts, Ecad-positive and GATA3-negative cells as nephrons, and PDGFRb-positive cells as renal stroma were counted under the same magnification, and the percentage (%) of GFP-positive cells in each of the three lineages was calculated by dividing the number of GFP-positive cells by the total number of cells. Two randomly selected sections from the renal organoid specimen were photographed at the same magnification, and a total of six images of each were made for analysis. This work was performed by two researchers.

### Measurement of the Number of CD3 Positive Cells Per Unit Area

Immunostained sections of each frozen organoid were photographed with a fluorescence microscope. The number of CD3-positive cells per unit area (pcs/mm^2^) was calculated by counting the total number of CD3-positive T lymphocytes under the same magnification and dividing the total number of cells by the area. Two randomly selected sections from two specimens of renal organoids were photographed at the same magnification, making a total of six images each for analysis. This work was performed by two researchers.

### Measurement of the Number of Nephrons Per Unit Area

HE-stained sections of organoid specimens of each cell ratio were photographed under the all-in-one fluorescence microscope to determine the number of glomeruli and area of organoids. The number of glomeruli per unit area (pcs/mm^2^) was then calculated by dividing the number of glomeruli by the area of the organoid. Two specimens from each group of renal organoids of each cell ratio were taken, and three randomly selected sections were photographed at the same magnification for a total of six images in each group for subsequent analysis. This measurement process was performed by two researchers.

### Statistical Analysis

Data were expressed as the mean ± standard error of the mean and analyzed using the Kruskal–Wallis test with a *post-hoc* test for comparison. All statistical analyses were performed using the Prism 8 software, and a p-value of <0.05 was considered significant.

## Results

### *In Vitro* Reconstruction of the Nephrogenic Niche in Interspecies Chimeric Renal Organoids

**Figure 1:**
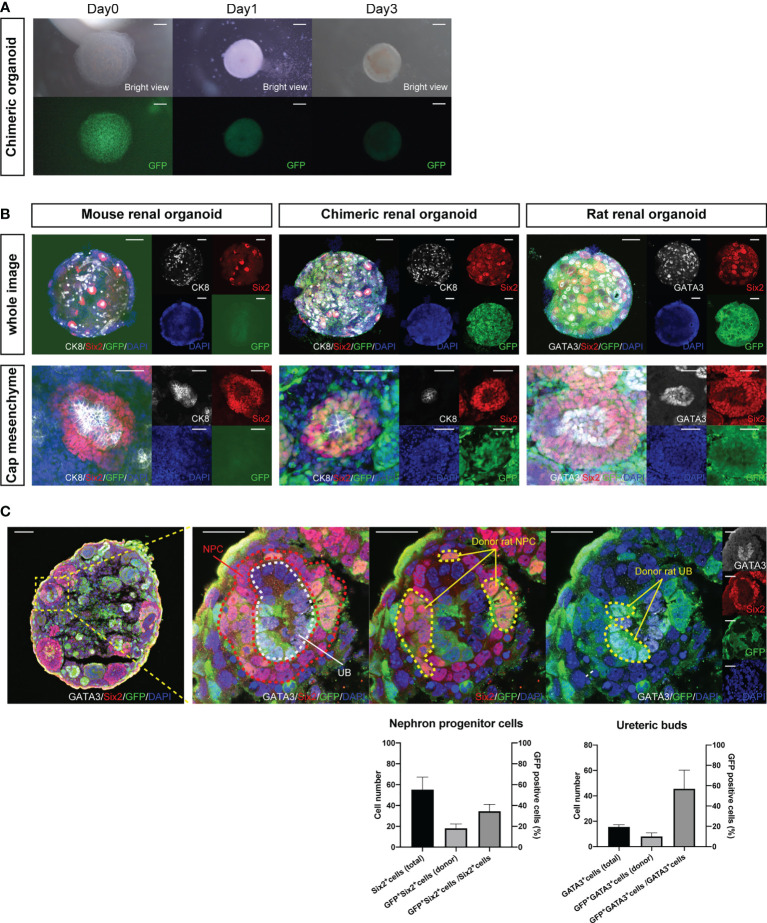
Reconstructed nephrogenic niche in mouse-rat heterologous chimeras. **(A)** Images of changes over time in reaggregated spheroids of 1:1 mixtures of mouse and GFP rat fetal kidney cells, which aggregated into spheroids on Day 1. The cells aggregated into spheroids on Day 1. The expression of GFP in the spheroids was uniform and not polarized (scale bars 100 μm). **(B)** Whole-mount immunostaining of **(A)**. Multiple six2-positive cap mesenchymes were observed in mouse renal organoids, chimeric renal organoids, and rat renal organoids (scale bars in the upper column: 200 μm). In mouse renal organoids, six2-positive and GFP-negative NPCs reaggregated around CK8-positive and GFP-negative UBs. In rat renal organoids, six2-positive and GFP-positive NPCs aggregated around GATA3-positive and GFP-positive UBs. In chimeric renal organoids, both six2-positive NPCs and CK8-positive UBs were composed of GFP-positive rat cells and GFP-negative mouse cells (scale bars in the lower column: 50 μm). **(C)** Immunostaining of cryosections of chimeric renal organoids. Cap mesenchyme consisting of Six2-positive NPCs (red dotted line), GATA3-positive UBs (white dotted line) was reconstructed, and GFP-positive and Six2-positive donor rat NPCs, GFP-positive and GATA3-positive donor rat UBs were observed (yellow dotted line) (scale bar, left column: 50 μm, right column: 20 μm). The ratio of GFP-positive and Six2-positive cells to all Six2-positive cells and the ratio of GFP-positive and GATA3-positive cells to all GATA3-positive cells at the same magnification are shown as percentages. Error bars in the graphs represent the standard error of the mean (n=6).

Fetal kidneys of B6 mice and GFP rats were subjected to enzymatic treatment to obtain single cells. Single mouse cells were mixed with single rat cells in the same proportion to create chimeric cell aggregates *via* centrifugation on Day 0. On Day 1, chimeric spheroids of B6 mice and GFP rats were formed (**Figure 1A**). Next, we observed whether the nephrogenic niche of the interspecies chimeric renal organoids was reconstructed by whole-mount immunostaining on Day 3 (**Supplemental Figure 1**). In the renal organoids of mice only, a cap mesenchyme was reconstructed in which six2-positive and GFP-negative mouse NPCs aggregated around cytokeratin 8 (CK8)-positive and GFP-negative mouse UBs (**Figure 1B**, left). In GFP rat-only organoids, cap mesenchyme was reconstructed with six2-positive and GFP-positive rat NPCs aggregated around GATA3-positive and GFP-positive rat UBs (**Figure 1B**, right). In the mouse-rat chimeric renal organoids, the cap mesenchyme was also reconstructed, and NPCs and UBs were composed of GFP-negative mouse cells and GFP-positive rat cells in a mosaic pattern (**Figure 1B**, middle). In addition, immunostaining of frozen sections was used to verify the extent to which GFP-positive rat renal progenitor cells contributed to the cap mesenchyme. The total number of Six2-positive NPCs was 55.2 ± 12.1, the number of GFP-positive and Six2-positive NPCs was 18.0 ± 4.3, and the GFP-positive rate of NPCs was 34.5 ± 6.7%. The total number of GATA3-positive UB cells was 15.5 ± 1.7, the number of GFP-positive and GATA3-positive UB cells was 8.0 ± 2.8, and the GFP-positive rate of UB was 45.6 ± 14.6% (**Figure 1C**).

### *In Vivo* Differentiation of Interspecies Chimeric Renal Organoids Into Three Renal Progenitor Cell Lineages

**Figure 2:**
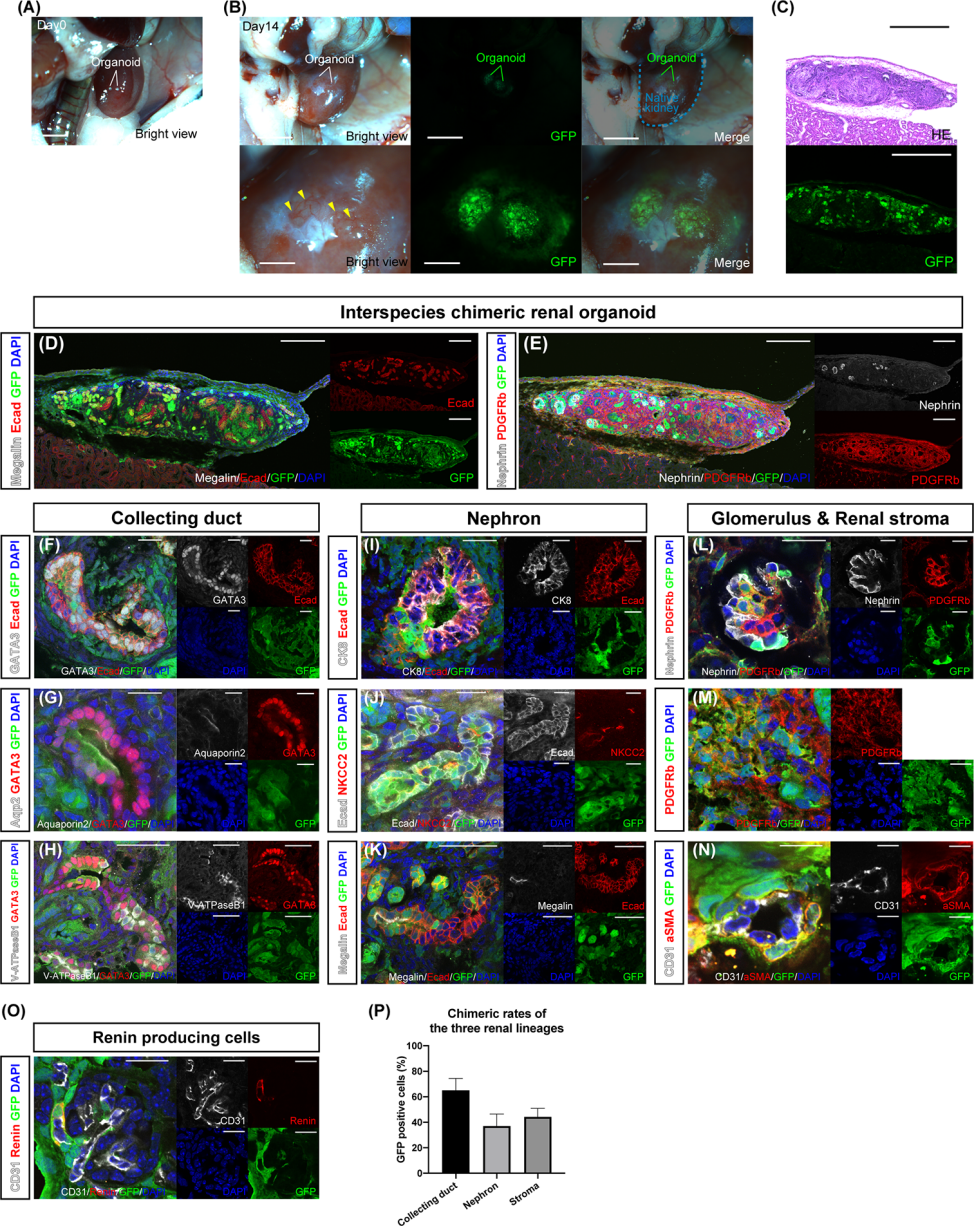
*In vivo* differentiation of chimeric renal organoids into renal tissue. **(A)** Images of transplanted chimeric renal organoids made from mouse and rat fetal kidneys under the renal capsule of immunodeficient mice (scale bars 2 mm). **(B)** Brightfield and fluorescence images of chimeric renal organoids 14 days after transplantation (scale bars, upper column: 1 mm, lower column: 1 mm). Recipient-derived blood vessels invade the organoid (yellow arrowhead). **(C)** Fluorescence microscopy and HE staining images of frozen sections (scale bar 500 μm). **(D)** Fluorescence immunostaining image of a frozen section of the whole renal organoid (scale bar 200 μm). Multiple Ecad-positive tubular structures. **(E)** Nephrin-positive glomeruli and PDGFRb-positive renal stroma in the organoid (scale bar 200 μm). **(F)** GATA3-positive and Ecad-positive collecting ducts consisted of GFP-positive rat cells and GFP-negative mice cells (scale bar 20 μm). **(G)** Aquaporin 2-positive and GATA3-positive principal cells consisted of GFP-positive rat cells and GFP-negative mice cells (scale bar 20 μm). **(H)** V-ATPaseB1-positive and GATA3-positive intercalated cells consisted of GFP-positive rat cells and GFP-negative mice cells (scale bar 50 μm). **(I)** Connection of GFP-negative and CK8-positive mouse collecting ducts to GFP-positive and Ecad-positive rat distal tubules (scale bar 20 μm). **(J)** NKCC2-positive loop of Henle consisting of GFP-positive rat cells and GFP-negative mice cells (scale bar 20 μm). **(K)** GFP-positive and megalin-positive proximal tubules connected to the tubules of GFP-negative mice cells (scale bar 50 μm). **(L)** Nephrin-positive podocytes and PDGFRb-positive mesangial cells generated from GFP-positive rat cells and GFP-negative mice cells in the glomeruli (scale bar 20 μm). **(M)** PDGFRb-positive interstitial fibroblasts consisted of GFP-positive rat cells and GFP-negative mice cells (scale bar 20 μm). **(N)** α SMA-positive vascular pericytes from GFP-positive rat cells (scale bar 10 μm). **(O)** Renin-producing cells were found around the afferent arterioles of the glomeruli (scale bar 20 µm). **(P)** Contribution of GFP-positive rat cells to the three lineages of collecting ducts, nephrons, and stroma. The percentages of GFP-positive cells in GATA3-positive collecting ducts, Ecad-positive, and GATA3-negative nephron tubules, and PDGFRb-positive stroma are shown. Error bars in the graphs represent the standard error of the mean (each group, n = 6).

Chimeric renal organoids—a mixture of single cells from the fetal kidneys of B6 mice and GFP rats in the same proportion—were transplanted under the renal capsule of adult NOG mice—an *in vivo* environment—for further maturation (**Figure 2A** and **Supplemental Figure 2**). The specimens were collected on day 14 because the chimeric organoids gradually became disrupted by their own hydronephrosis after long-term transplantation beyond day 14. At the time of retrieval after 14 days, the chimeric renal organoids expressed GFP and were invaded by GFP-negative blood vessels derived from the recipient mice (**Figure 2B**, yellow arrowhead). Cryosections of the chimeric renal organoids showed extensive GFP expression, and HE staining revealed luminal structures (**Figure 2C**). Moreover, fluorescence immunostaining of the cryosections showed that the whole chimeric renal organoids comprised GFP-positive rat cells and GFP-negative mouse cells, with Ecad-positive tubules (**Figure 2D**), nephrin-positive glomeruli, and PDGFRb-positive renal stroma (**Figure 2E**). In GATA3-positive and Ecad-positive collecting ducts comprising GFP-positive rat cells and GFP-negative mouse cells were also observed (**Figure 2F**). Aquaporin 2-positive, GATA3-positive, GFP-positive rat principal cells, (**Figure 2G**) and V-ATPaseB1-positive, GATA3-positive, and GFP-positive rat intercalated cells (**Figure 2H**) were observed, forming luminal structures with GFP-negative mouse cells. Ecad-positive and GFP-positive rat distal tubules were connected to the GFP-negative and CK8-positive mouse collecting ducts (**Figure 2I**). An NKCC2-positive loop of Henle comprising GFP-positive rat cells and GFP-negative mouse cells was observed (**Figure 2J**). A megalin-positive and GFP-positive proximal tubule forming a lumen with GFP-negative mouse cells was also observed (**Figure 2K**). In the nephrin-positive glomeruli, podocytes comprised GFP-positive rat cells and GFP-negative mouse cells and PDGFRb-positive mesangial cells comprised GFP-positive rat cells and GFP-negative mouse cells (**Figure 2L**). PDGFRb-positive interstitial fibroblasts also comprised GFP-positive rat cells and GFP-negative mouse cells (**Figure 2M**). Furthermore, α SMA-positive vascular pericytes comprising GFP-positive rat cells were observed (**Figure 2N**). In addition, as proof of organoid functionality, we observed GFP-positive renin-producing cells with endocrine functions (**Figure 2O**). We also examined the contribution of GFP-positive rat cells to each of the three lineages by counting the cells that were immunostained for representative cell markers. The percentage of GFP-positive cells in GATA3-positive collecting ducts was 66.1 ± 9.3%, that of GFP-positive cells in the tubules of Ecad-positive and GATA3-negative nephrons was 37.1 ± 9.4%, and that of GFP-positive cells in PDGFRb-positive stroma was 44.2 ± 6.7% (**Figure 2P**).

### Reduction of Xenogeneic Rejection in Interspecies Chimeric Renal Organoid Transplantations

**Figure 3:**
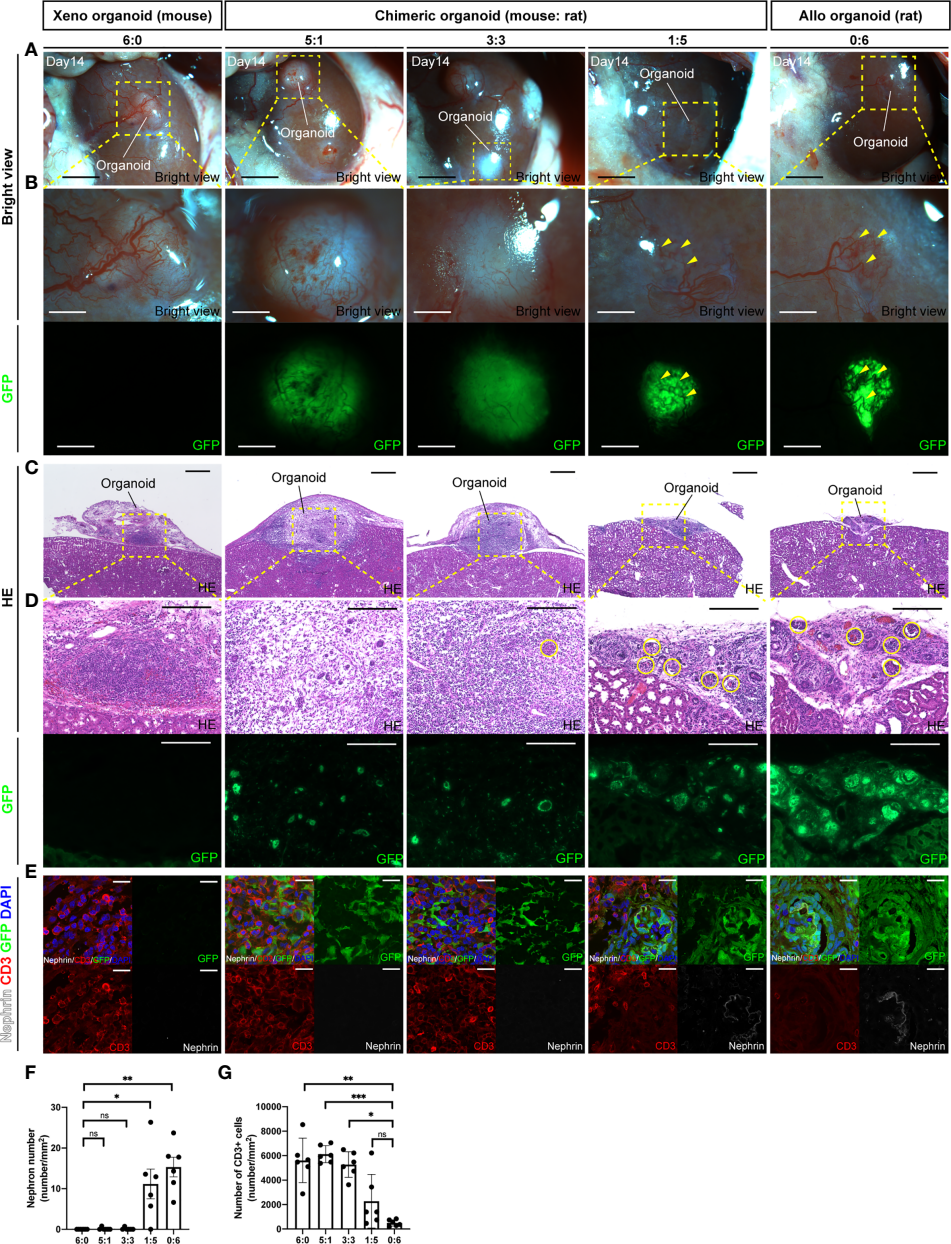
Chimeric renal organoids reduce xenograft rejection. **(A)** Images of chimeric renal organoids generated from single cells of mouse and rat fetal kidneys at different cell ratios at 14 days after transplantation into the renal capsule of SD rats under mild immunosuppression protocol (scale bar 1 mm). **(B)** Magnified image of **(A)**. The 6:0 xenograft mouse renal organoids and the 5:1 and 3:3 chimeric renal organoids were white and enlarged, and the expression of GFP in the chimeric organoids was weak. The 1:5 chimeric kidney organoids showed the same vascular invasion of the recipient (yellow arrowhead) and strong GFP expression as the allogenic 0:6 rat renal organoids, the control (scale bars, upper column: 1 mm, lower column: 1 mm). **(C)** HE-stained images of each renal organoid. The 6:0 mouse renal organoids and 5:1 and 3:3 chimeric renal organoids were thickened and enlarged (scale bar 500 μm). **(D)** Magnified images of HE staining and GFP expression in each renal organoid. Both the xenogeneic mouse renal organoids and the chimeric renal organoids showed inflammatory cell infiltration and only a portion of the glomerulus remained (yellow circles). The 1:5 chimeric kidney organoids showed mild inflammatory cell infiltration and multiple glomeruli as in the control allogenic rat renal organoids (scale bars, upper column: 200 μm, lower column: 200 μm). **(E)** Images of CD3 immunostaining in each renal organoid. Xenograft 6:0 renal organoids, 3:3, and 5:1 chimeric renal organoids showed many CD3-positive cells and few glomerular structures. In 1:5 chimeric renal organoids, CD3-positive cells mainly infiltrated the region composed of GFP-negative heterologous mice, whereas renal tissue composed of GFP-positive rat cells showed less infiltration of CD3-positive cells (scale bar 20 μm). **(F)** The 1:5 chimeric renal organoids with a higher rat cell ratio had significantly higher glomerular counts compared to the 6:0 xenograft renal organoids (n = 6 in each group). Data are expressed as the mean ± standard error of the mean (Kruskal–Wallis test with *post-hoc* test). **(G)** The 1:5 chimeric renal organoids with a high rat cell ratio showed no significant difference in the number of CD3-positive cells compared to the 0:6 allogeneic renal organoids, but the 3:3, 5:1 chimeric renal organoids, and 6:0 xenograft renal organoids had significantly higher numbers of CD3-positive cells (n = 6 for each group). Data are expressed as the mean ± standard error of the mean (Kruskal–Wallis test with *post-hoc* test). *P < 0.05; **P < 0.01; ***P < 0.001; ns, not significant.

To investigate whether chimeric renal organoids reduce rejection compared with xenograft organoids, renal organoids were prepared *in vitro* by mixing cells from the fetal kidneys of B6 mice and SD rat-based GFP rats in various ratios (6:0, 5:1, 3:3, 1:5, and 0:6) and transplanted into the renal capsule of adult SD rats. The recipients were subjected to mild immunosuppression (tacrolimus 0.3 mg/kg/day) and organoids of the respective cell ratios were retrieved at 14 days after transplantation (**Supplemental Figure 3**). At the time of retrieval, 6:0 xenograft mouse renal organoids and 5:1 and 3:3 chimeric renal organoids were white and swollen, whereas 1:5 chimeric renal organoids and 0:6 allogeneic rat renal organoids were not swollen, showing vascular invasion from the recipient rat and strong GFP expression (**Figures 3A, B**). HE staining of the same tissue showed that the 6:0 xenograft mouse renal organoids and the 5:1 and 3:3 chimeric renal organoid tissues were thick and swollen with strong inflammatory cell infiltration and few glomeruli (**Figures 3C, D, F**). However, the 1:5 chimeric renal organoids, in which the cell ratio of the same rat as the recipient was increased, showed mild infiltration of inflammatory cells and mild abolition of glomeruli by inflammatory cells (**Figures 3C, D, F**). Furthermore, inflammatory infiltration was evaluated by immunostaining for CD3. Infiltration of CD3-positive cells was observed mainly in areas composed of xenogeneic, GFP-negative mouse cells, whereas areas generated from GFP-positive rat cells showed less infiltration of CD3-positive cells, and the remaining glomeruli were composed mainly of GFP-positive rat cells (**Figure 3E**). The number of CD3-positive cells per unit area was not significantly different in the 1:5 chimeric renal organoids compared to the control 0:6 allogeneic rat renal organoids, but the number of CD3-positive cells was significantly increased in the 6:0 xenograft renal organoids and the 5:1 and 3:3 chimeric renal organoids (**Figure 3G**).

## Discussion

In this study, we confirmed the chimeric establishment of rat renal progenitor cells within the mouse nephrogenic niche *in vitro* by mixing single cells of interspecies (mouse-rat) fetal kidneys. Furthermore, in the *in vivo* environment of immunodeficient mice, the mouse nephrogenesis mechanism could act on rat renal progenitor cells to generate chimeric renal organoids containing mature nephrons, collecting ducts, and stroma in a mosaic pattern comprising mouse and rat cells. We also demonstrated that increasing the ratio of recipient-derived renal cells in chimeric renal organoids attenuated rejection.

*In vitro* chimeric renal organoids showed that NPCs and UBs, as well as SPCs surrounding NPCs, comprised GFP-positive rat cells and GFP-negative mouse cells (**Figure 1B**, middle). Transplantation into the ipsilateral renal membrane of immunodeficient mice allowed the generation of chimeric renal tissue containing all three lineages (**Figures 2D**–**N**). This result was consistent with that of studies on cell lineage analysis showing that NPCs differentiate into podocytes, proximal tubules, and distal tubules (13) and SPCs differentiate into interstitial fibroblasts, mesangial cells, and vascular pericytes (14) as well as of a study showing that UBs differentiate into collecting ducts (15). Recently, the generation of human renal organoids containing nephrons, renal stroma, and collecting ducts from human iPSCs *in vitro* using a single protocol has been reported (16). However, faithful reproduction of the renal developmental mechanism and human kidney from human iPSCs remains challenging (17). In the future, a method should be developed to accurately induce individual differentiation into each progenitor cell such as NPC, UB, and SPC and to subsequently induce each lineage into renal tissue. NPCs and UBs differentiated from human mesenchymal stem cells or human iPSCs can reportedly be mixed with single cells from fetal mouse kidneys to facilitate the crossing of the heterogeneous barrier and differentiation into single lineage tissues such as nephrons and collecting ducts (18–20). Our results showed that not only differentiation into a single lineage but also the simultaneous interspecies generation of all three progenitor cell lineages of renal tissue, including the stroma, is possible. Because the gap in developmental signals is considered relatively small even between different species during organogenesis at the time of fetal kidney formation (21, 22), the present phenomenon indicates that signals related to the nephrogenesis of NPCs, UBs, and SPCs can be shared among different species. These results indicate that animal fetal kidneys have the ability to generate mature renal tissue between different species, which can be used for organ regeneration.

We previously verified suitable transplantation sites for the development of fetal and adult kidneys. For fetal kidney transplantation, we have shown that the periaortic region is superior for development in a small animal model of rats (23). In preclinical xenotransplantation of adult kidneys from pigs to monkeys, we have shown that orthotopic transplantation is superior (24). For the transplantation of renal organoids, we have previously developed a simple method of transplanting them under the renal membrane without damage (10). In the present study, we transplanted them under the renal capsule of the host and could generate mature renal tissues between different species.

Xenotransplantation has been garnering attention as a promising solution to the shortage of transplanted organs. It has already been demonstrated that long-term life support is possible in porcine-to-primate kidney transplantation (25). However, because strong immunosuppression is sometimes required to cause infection and death of the recipient, modification of the recipient by bone marrow chimeras (26) and attempts to reduce immunogenicity are required for clinical application in humans (27). The results of the present study show that chimeric renal organoids with a high engraftment cell fraction have immunological advantages compared with complete heterologous organoids (**Figures 3A**–**E**). Regarding this chimeric phenomenon and attenuation of immune responses, a previous study has described a mouse model that showed the possibility of dispersing the number of reactive T-cell precursors on the recipient side by transplanting islet cells derived from multiple donors (28) and a mouse model that leans the recipient’s bone marrow cells toward the donor side by bone marrow transplantation (29). Furthermore, the phenomenon of reduced rejection while receiving immunosuppressive drugs has been clinically explained by the establishment of microchimerism (30). The fetal kidneys used as the cell source in this study have low expression of donor antigens that are subject to xenotransplant rejection (31). When used for transplantation, the vasculature is supplied by the recipient (32). In the present chimeric renal organoids, the vasculature also entered from the periphery (**Figures 2B**, **3A, B**), which may reduce their immunogenicity compared with that of normal adult kidney transplantation. From this immunological perspective, we are now focusing on the generation of chimeric kidneys with a higher chimerism rate derived from donor cells not only for the nephron, which can be regenerated by replacement of progenitor cells (8), but also for the renal stroma (9), collecting ducts, and ureter.

There are limitations to this study. First, this chimeric renal organoid is a closed system, and it will gradually be disintegrated by its own urine just like common renal organoids. Therefore, research on the connection to functional urinary tract components such as the ureter and bladder are necessary in the future. Next, in this study, mice and rats were relatively closely related animals for the chimera experiment. Pigs and humans, which we are considering in the future, are more distant from each other, and it may be difficult to create chimeras like this one. However, in addition to reports of the generation of interspecies chimeras by editing apoptotic genes into donor human cells (33), there are also reports of increased interspecies chimera rates by editing cell division and apoptosis-related genes into host cells (34), which may be applicable to our chimera technology.

Currently, we are investigating the generation of interspecies chimeric renal organoids using renal progenitor cells derived from human iPS cells and rodents that are more closely related species to each other than pigs. Once human-animal chimeric renal organoids are generated and mature human renal tissues can be generated from renal progenitor cells, they can be used not only for organ transplantation but also for various clinical applications such as drug sensitivity testing, disease models, and drug discovery using patient-derived or gene-edited iPS cells.

In conclusion, renal developmental niches were reconstructed between mouse and rat heterologous species, each capable of differentiating into renal tissue of three renal progenitor cell lineages. Furthermore, compared with xenotransplantation, interspecies chimeric renal organoids with a high chimerism rate of allogeneic cells could reduce xenogeneic rejection. These results will not only serve as a basis for future chimera technology to generate hybrid kidneys containing human renal tissue using animal fetal nephrogenesis but also help advance research on xenotransplantation and stem cells.

## Data Availability Statement

The original contributions presented in the study are included in the article/**Supplementary Material**. Further inquiries can be directed to the corresponding author.

## Ethics Statement

The animal study was reviewed and approved by The Institutional Animal Care and Use Committee and the Safety Committee for Genetic Recombination Experiments of The Jikei University School of Medicine.

## Author Contributions

YS, EK, SY, and TY designed the study. YS, with the help of NM, carried out the experiments. YS, with the help of NM, collected and analyzed the data. YS, with the help of EK and SY, wrote the manuscript. EK, SY and TY supervised the execution of the study. All authors contributed to the article and approved the submitted version.

## Funding

This work was supported by the Japan Agency for Medical Research and Development (AMED; grants 21bk0104094h0003 and 21bm0704049h0002), the Japan Society for the Promotion of Science (JSPS-KAKENHI; grants 21K08288), and grants for pathophysiological research conference in chronic kidney disease (grants JKFB21-2).

## Conflict of Interest

The authors declare that the research was conducted in the absence of any commercial or financial relationships that could be construed as a potential conflict of interest.

## Publisher’s Note

All claims expressed in this article are solely those of the authors and do not necessarily represent those of their affiliated organizations, or those of the publisher, the editors and the reviewers. Any product that may be evaluated in this article, or claim that may be made by its manufacturer, is not guaranteed or endorsed by the publisher.

## Supplementary Material

Supplementary Figure 1 Schematic diagram of the experiments associated with **Figure 1**. *In vitro* generation of nephrogenic niches of interspecies chimeric renal organoids created by enzymatic treatment of fetal B6 mouse kidneys and fetal GFP rat kidneys into single cells, each mixed in equal proportions. RPC, renal progenitor cell.

Supplementary Figure 2 Schematic diagram of the experiments associated with **Figure 2**. *In vivo* maturation of interspecies chimeric renal organoids under the renal capsule of immunocompromised mice, created by mixing fetal B6 mouse kidney and fetal GFP rat kidney into a single cell by enzymatic treatment, each in the same proportion.

Supplementary Figure 3 Schematic diagram of the experiments associated with **Figure 3**. Renal organoids mixed with cells from the fetal kidneys of B6 mice and GFP rats in various ratios (6:0, 5:1, 3:3, 1:5, and 0:6) were prepared *in vitro*, transplanted into SD rats under mild immunosuppression, and the grafts were collected 14 days later.
