# Supplementary figures and images for: Beneficial Impact of Interspecies Chimeric Renal Organoids Against a Xenogeneic Immune Response
Source: Front Immunol. 2022 Feb 15;13:848433. doi: 10.3389/fimmu.2022.848433 (PMC8885510; doi:10.3389/fimmu.2022.848433)

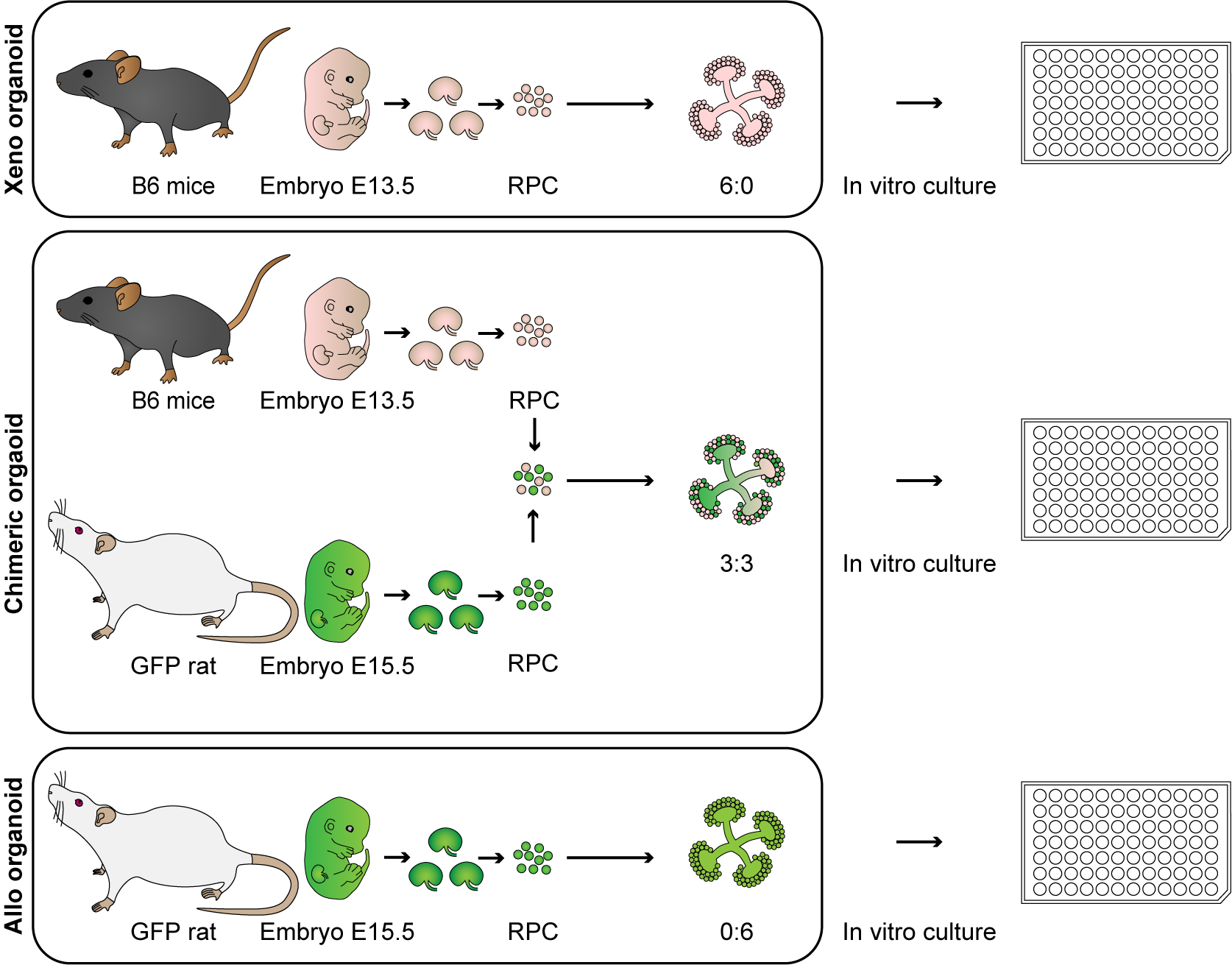

Supplement: Supplementary Figure 1 — Schematic diagram of the experiments associated with Figure 1 . In vitro generation of nephrogenic niches of interspecies chimeric renal organoids created by enzymatic treatment of fetal B6 mouse kidneys and fetal GFP rat kidneys into single cells, each mixed in equal proportions. RPC, renal progenitor cell. [file Image_1.tif]

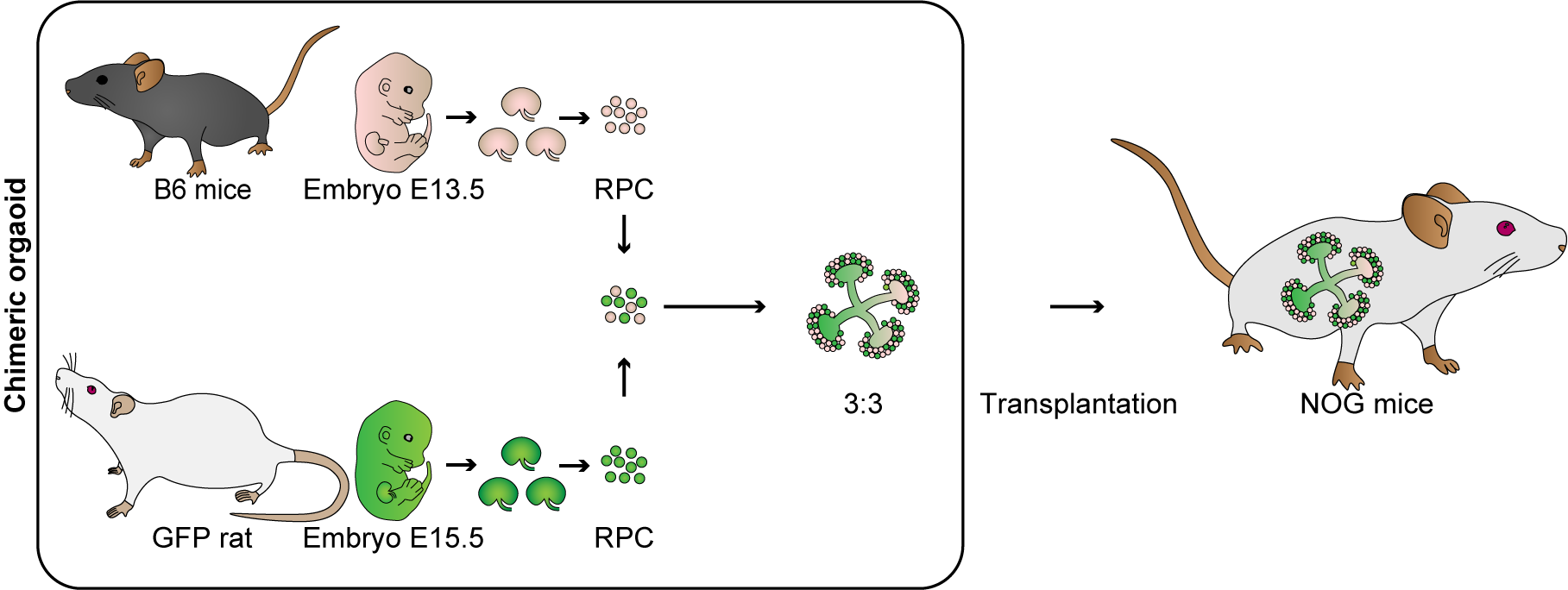

Supplement: Supplementary Figure 2 — Schematic diagram of the experiments associated with Figure 2 . In vivo maturation of interspecies chimeric renal organoids under the renal capsule of immunocompromised mice, created by mixing fetal B6 mouse kidney and fetal GFP rat kidney into a single cell by enzymatic treatment, each in the same proportion. [file Image_2.tif]

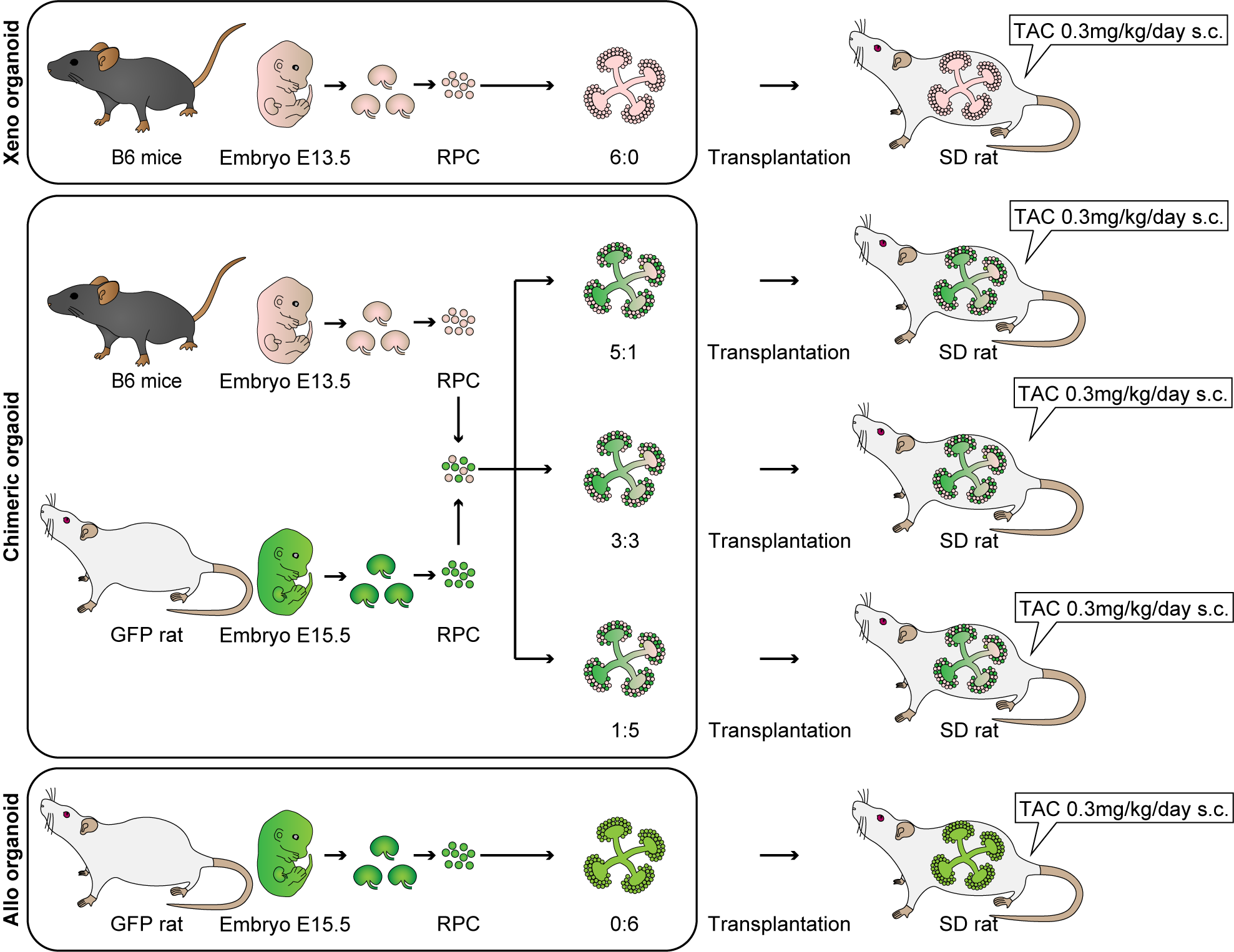

Supplement: Supplementary Figure 3 — Schematic diagram of the experiments associated with Figure 3 . Renal organoids mixed with cells from the fetal kidneys of B6 mice and GFP rats in various ratios (6:0, 5:1, 3:3, 1:5, and 0:6) were prepared in vitro, transplanted into SD rats under mild immunosuppression, and the grafts were collected 14 days later. [file Image_3.tif]
